# Neurophysiology in psychosis: The quest for disease biomarkers

**DOI:** 10.1038/s41398-022-01860-x

**Published:** 2022-03-11

**Authors:** Baihan Wang, Eirini Zartaloudi, Jennifer F. Linden, Elvira Bramon

**Affiliations:** 1 Division of Psychiatry, University College London, London, UK; 2 Institute of Clinical Trials and Methodology, University College London, London, UK; 3 Ear Institute, University College London, London, UK; 4 Department of Neuroscience, Physiology & Pharmacology, University College London, London, UK; 5 Institute of Cognitive Neuroscience, University College London, London, UK

**Keywords:** Biomarkers, Bipolar disorder, Schizophrenia, Neuroscience

## Abstract

Psychotic disorders affect 3% of the population at some stage in life, are a leading cause of disability, and impose a great economic burden on society. Major breakthroughs in the genetics of psychosis have not yet been matched by an understanding of its neurobiology. Biomarkers of perception and cognition obtained through non-invasive neurophysiological tools, especially EEG, offer a unique opportunity to gain mechanistic insights. Techniques for measuring neurophysiological markers are inexpensive and ubiquitous, thus having the potential as an accessible tool for patient stratification towards early treatments leading to better outcomes. In this paper, we review the literature on neurophysiological markers for psychosis and their relevant disease mechanisms, mainly covering event-related potentials including P50/N100 sensory gating, mismatch negativity, and the N100 and P300 waveforms. While several neurophysiological deficits are well established in patients with psychosis, more research is needed to study neurophysiological markers in their unaffected relatives and individuals at clinical high risk. We need to harness EEG to investigate markers of disease risk as key steps to elucidate the aetiology of psychosis and facilitate earlier detection and treatment.

## Introduction

Psychotic disorders, including schizophrenia and bipolar disorder, have a lifetime prevalence of ~3% [1] and are the leading cause of disability in young adults [2, 3]. They have a major economic impact, with an annual cost of €94 billion for psychotic disorders across Europe [4] and $156 billion for schizophrenia in the United States [5]. Major breakthroughs have been made in our understanding of the environmental and genetic origins of psychosis, with 270 loci associated with schizophrenia and several compelling rare genetic variants identified so far [6–8]. There are also a range of effective treatments for psychosis, including over 20 different drugs licensed for schizophrenia [9], as well as psychological therapies and rehabilitation interventions [10, 11]. However, the psychoses are highly heterogeneous in their clinical presentations and our understanding of underlying mechanisms remains limited. We face several challenges, including substantial delays in treatment access (typically of 6–12 months in the UK) [12], as well as low adherence to antipsychotics, with over 50% of people stopping their medication within two years for a variety of reasons, including adverse drug reactions [13, 14].

A potential solution to these challenges involves studying biomarkers for psychosis seeking to unravel its underlying mechanisms and develop new treatments. Biomarkers are quantitative traits that can be objectively and reliably measured [15], typically using laboratory, imaging, or standardised clinical assessment tools. Since psychosis is characterised by perceptual and cognitive deficits that often manifest prior to the onset or diagnosis of the illness [16], biomarkers of brain functions in relation to information processing are particularly attractive in psychosis research. Neurophysiological tools, especially electroencephalography (EEG) and event-related potentials (ERPs, also known as evoked potentials), which are changes in the EEG triggered by stimuli, are promising due to their exceptional temporal resolution that allows the study of perception and cognition safely in vivo. They are relatively inexpensive and ubiquitous, thus permitting the collection of large samples required for progress in understanding the aetiology of psychosis. Once validated in patients, such EEG/ERP biomarkers could pinpoint neurocognitive mechanisms most relevant to psychosis, and thus serve as targets for the development of new treatments.

There has been growing interest in neurophysiological biomarkers of psychosis risk, often studied in unaffected relatives of patients or individuals at clinical high risk. Consistent with the observation that heritability estimates for psychotic disorders are about 80% [17], some neurophysiological markers are also impaired in the unaffected relatives of patients with psychosis. Such biomarkers, also referred to as endophenotypes, can help as intermediate phenotypes to explain how the genetic risk of psychosis is conferred [18]. Several neurophysiological markers have also been found to be associated with increased psychosis risk amongst individuals at clinical high risk, or even predictive of transition to psychosis [19, 20]. Their implementation in assessments could help facilitate earlier and personalised treatment, thus leading to improved prognosis [21].

In this paper, we review the vast literature on neurophysiological markers that have been found to be impaired in patients with psychosis. In addition, we summarise the neuropsychological and clinical correlates of those biomarkers and discuss the insights they can offer into the underlying neurobiology of psychosis. We also review evidence for neurophysiological markers of disease risk reported in the unaffected relatives of patients and individuals at clinical high risk for psychosis, which are important for both mechanistic and clinical research in psychosis.

## P50 and N100 sensory gating

The P50 waveform is a positive voltage ERP, a change in the EEG triggered by stimuli, which is used to measure sensory gating [22, 23]. The classic experiment to measure P50 sensory gating is the dual-click paradigm, where participants are presented with pairs of auditory stimuli (usually separated by 500 ms) and the amplitude of the ERP at approximately 50 ms after each stimulus constitutes the P50 [22]. The first stimulus is labelled the conditioning stimulus (C), the second stimulus is labelled the test stimulus (T), and P50 sensory gating is measured as the T/C ratio [24]. A ratio below 50% is usually considered a normal P50 sensory gating response [25]. Although studies on sensory gating in psychosis were primarily conducted on the P50, there has been increasing research recently on the N100 response. While the P50 reflects the early pre-attentive stage of sensory gating, the N100 involves later stages of information processing that share similar gating mechanisms.

Extensive evidence shows that individuals with psychotic disorders display larger responses to the second stimulus compared to healthy controls, with only a 20–50% suppression, resulting in increased P50 T/C ratios [22, 23, 26, 27]. The latest meta-analysis of P50 sensory gating reported that compared to controls (*n* = 3464), people with schizophrenia (*n* = 3666) and bipolar disorder (*n* = 656) had higher P50 ratios (Cohen’s *d* = 10.30 and 4.51, respectively) [24], indicative of impaired suppression. By contrast, there has been less research on N100 sensory gating in psychosis. A recent meta-analysis including 1027 patients with schizophrenia and 1131 controls found that the bigger T/C ratio of N100 in patients was likely due to their reduced amplitude to the conditioning stimuli (Hedges’ *g* = −0.61), instead of insufficient suppression of the test stimuli (Hedges’ *g* = −0.04) [28]. Although this meta-analysis revealed N100 reductions, these were not due to gating deficits in schizophrenia.

Reduced P50 gating reflects deficits in inhibitory processes and/or sensory adaptation, which could contribute to the sensory overload some patients experience. The few studies investigating the clinical correlates of P50 gating showed little evidence of association with psychotic symptoms but instead reported correlations between P50 gating and attention [29–32]. Thus, the research shows that sensory gating is not so much related to symptoms, but rather to the cognitive dysfunction that characterises psychosis.

P50 sensory gating has been associated with the CA3 region of the hippocampus in rodent models, as shown by studies using intracranial electrodes to investigate the analogous P20/N40 gating in rats [33]. In addition, human studies also suggested the involvement of the thalamus, the temporal cortex, and the frontal cortex in the generation of P50 sensory gating [34, 35]. Since P50 sensory gating is mainly mediated by neural oscillations in gamma and beta frequencies [36–38], it has been associated with the network of pyramidal neurons and GABAergic interneurons. It is hypothesised that when activated by auditory input, the interneurons release GABA that inhibits the activity of the pyramidal neurons, and thus diminishes the response to the second stimulus [39, 40]. P50 sensory gating has been proposed as a target for translational research, and the effect of pharmacological treatments on P50 sensory gating, especially drugs acting on nicotinic receptors, were found to be comparable across animal and human studies [39, 41].

P50 sensory gating impairment is also associated with the genetic risk of psychosis, making it a psychosis endophenotype. Twin studies of P50 sensory gating showed heritability estimates of about 68% [42], and increased P50 ratios have been found in the unaffected relatives of patients with psychosis (Cohen’s *d* = 2.26; 769 relatives and 3464 controls) [24]. Therefore, efforts have been made to explore the genetic basis of P50 sensory gating, as a strategy to elucidate biological processes relevant in psychosis. In line with the findings from pharmacological research, early candidate-gene and linkage analysis found that P50 sensory gating was associated with two α7 nicotinic acetylcholine receptor subunit genes (CHRNA7 and CHRNA7-like genes) [43–45]. While this association awaits replication by the latest genome-wide and sequencing approaches, the α7 nicotinic acetylcholine receptor could be a target for drug development for psychosis [41].

A few studies investigated P50 and N100 sensory gating in individuals at clinical high risk for psychosis, but the results were less consistent [20, 46–48]. While some studies reported impaired P50 and N100 sensory gating in those individuals [49, 50], others did not find such differences [51, 52]. A meta-analysis comparing high-risk individuals who developed psychosis with those who did not become ill found no differences in P50/N100 sensory gating between the two groups, but only three studies were available for analysis thus limiting statistical power [19].

In conclusion, P50 sensory gating abnormalities are prominent in patients with psychosis. The relatives of patients also display consistent if milder P50 suppression deficits, which supports its role as a biomarker of genetic predisposition to psychosis, especially in relation to the α7 nicotinic acetylcholine receptor genetic variants. We have less evidence supporting P50 sensory gating as a marker of the clinical risk for psychosis. There is also limited research on N100 sensory gating in psychosis, and it remains to be determined if the observed deficits were truly impairments in sensory gating or, instead, in sensory registration.

## The N100 waveform as a marker of auditory processing

The N100 waveform, as a response to auditory stimuli, may also constitute a biomarker for psychosis. As the N100 can be easily elicited by any discernible auditory input regardless of task demand, various experimental designs have been used. The passive listening paradigm is perhaps the simplest way to measure the N100, where participants are presented with a series of identical auditory stimuli, and the N100 response is measured following each stimulus. Alternatively, in the auditory oddball task (widely used to measure the N100 and a range of ERPs), participants are asked to listen to a series of different auditory stimuli, including a small number of ‘deviant’ sounds randomly embedded in standard stimuli, which usually differ in pitch or duration [53]. The N100 can be observed following both the standard and the deviant stimuli, but the amplitude to the latter is consistently larger.

The literature comparing N100 between patients with psychosis and controls showed conflicting results [54]. However, N100 amplitude is strongly influenced by the interstimulus interval, with larger amplitudes observed in experiments using longer silent intervals [54, 55]. This reduces the statistical power of studies with short intervals to detect any group differences. Interestingly, this effect might also explain why the meta-analysis of N100 sensory gating only found decreased N100 amplitude to the first stimulus (C) but no changes to the second stimulus (T) in patients with schizophrenia, as only the first stimulus occurred after a long silent interval [28].

Although a review by Rosburg et al. concluded that the association between N100 amplitude and the general psychopathology of psychosis was weak [54], N100 deficits in patients could still reflect failures in specific domains of auditory processing related to the aetiology of psychosis. Given that the N1-P2 response complex can be used to measure hearing threshold, deficits in the N100 may well reflect subclinical hearing impairment. Indeed, hearing impairment is a risk factor for schizophrenia, with a pooled odds ratio of 3.15 in a meta-analysis of longitudinal studies [56]. Besides, some researchers suggested that N100 may reflect higher functions of the brain related to the detection of salient stimuli [57]. Patients with psychosis may have difficulty in detecting external stimuli due to the interference of internal stimuli [57], such as auditory hallucinations. Reduced N100 amplitude could also reflect a slower recovery of the N100 response in patients with psychosis compared to controls, which might explain why this impairment is more robustly detected at long interstimulus intervals [28].

The neurobiology of the N100 in relation to psychosis is not well understood. The N100 is mainly generated in the primary and association auditory cortices, as well as the frontal and motor cortices [58]. Some studies attempted to link the N100 to N-Methyl-d-Aspartate (NMDA) glutamate receptors function, which is implicated in the aetiology of psychosis [59]. However, the effect of NMDA receptor antagonists on the N100 is unclear, as previous studies investigating N40 in mice (the N100 analogous component in humans) reported mixed results [60, 61]. One study found that phencyclidine (an NMDA receptor antagonist) reduced N100 amplitude in monkeys at a long interstimulus interval [62], but another study reported that ketamine increased N100 amplitude in human participants [63].

In the oddball task, the heritability was estimated to be 60–70% for N100 amplitude [64, 65], and 56% for N100 latency at Cz [64]. However, it is unclear if the N100 response is impaired in the relatives of patients with psychosis due to limited and conflicting findings. While some studies reported reduced N100 amplitude or prolonged N100 latency in the unaffected relatives [66, 67], others did not find such differences [68]. Similarly, only a small number of studies have investigated N100 deficits amongst individuals at clinical high risk, with some reporting reduced N100 in clinical high-risk participants compared to controls [20, 50, 69]. In particular, one study found that a reduction in N100 amplitude between baseline and follow-up was only present in individuals at clinical high risk who made a transition to psychosis, but not in those without transition or controls [70].

The N100 remains under-researched in psychosis. Although reduced N100 amplitude at long interstimulus intervals has been reported in patients, whether or not the N100 is a marker for psychosis risk remains unclear, as there has been limited research in unaffected relatives and individuals at clinical high risk. Although N100 impairment is unlikely to be specific to psychosis [54], it offers a tool to elucidate the mechanisms through which deficits in auditory processing constitute a risk factor for psychosis. The literature covers a variety of experimental designs for the N100, usually obtained as a by-product in the oddball task, and we now need standardised paradigms targeted at a specific stage of auditory processing. An example of this is using N100 to measure corollary discharge, which is discussed in a later section in the review.

## The mismatch negativity

The mismatch negativity (MMN) is elicited using the auditory oddball paradigm, and appears as an increased negativity in the ERP evoked by deviant relative to standard stimuli around 150–250 ms after stimulus onset. No actions or responses are required and the MMN occurs without participants’ cooperation, allowing the investigation of the early pre-attentive stages of auditory processing.

Reduced MMN amplitude is a well-established finding in patients with psychosis [71–73]. In a meta-analysis, Erickson et al. summarised 104 studies on the MMN in various clinical groups [71]. They found evidence that patients with schizophrenia (*n* = 3797) had reduced MMN amplitude compared to controls (*n* = 3960) with a large effect size (Hedges’ *g* = −0.95), as well as a medium effect size for reduced MMN amplitude in patients with bipolar disorder (*n* = 240; Hedges’ *g* = −0.37) [71]. The MMN might also reflect disease progression in psychosis, as the effect size of MMN reduction in first-episode schizophrenia (Hedges’ *g* = −0.42) is only a half of that in chronic schizophrenia (Hedges’ *g* = −0.81) [71].

The reductions of MMN amplitude in schizophrenia are interpreted as evidence of predictive coding deficits in the auditory system [74]. The predictive coding theory postulates that the brain compares the sensory input and its internal representation (model) inferred from the previous inputs while trying to minimise the discrepancy between the two [75]. The MMN is thought to reflect the prediction error of the model based on standard stimuli when a deviant stimulus occurs [76]. This theory has been supported by dynamic causal modelling of experimental EEG data [77, 78], and neuronal models that simulated MMN based on NMDA receptor neurotransmission [79]. Patients with psychosis are hypothesised to have a faulty prediction processing system.

Another meta-analysis pooling 3485 patients did not find evidence for an association between symptom severity and MMN in schizophrenia [80], indicating that although MMN deficits reflect liability for schizophrenia, they may not be symptom dependent. Although the MMN is often described as a pre-attentive component, Damaso et al. proposed that the MMN might serve as a call for the following automatic allocation of attentional resources [81]. Patients with psychosis are known to have attention and other cognitive deficits, although this paper did not find consistent correlations between performance in MMN and neuropsychological tests of attention [81]. MMN has also been associated with the functional outcomes of psychosis [82, 83], indicating its potential utility as a marker for recovery and a target for treatment.

The MMN is mainly generated by the auditory and prefrontal cortices [72], and thought to be a physiological marker of NMDA glutamate receptor neurotransmission. There is converging evidence that NMDA receptor antagonists, such as ketamine, decrease MMN amplitude and increase MMN latency in human subjects, akin to changes observed in patients with psychosis [84]. Although links with NMDA function have been established, the neural mechanisms of the MMN are still not well understood. Since the MMN mainly maps into the theta waves (4–7 Hz), the somatostatin-type GABAergic interneurons linked to lower-frequency oscillations, as well as their interplay with the glutamatergic pyramidal neurons, have been proposed to play a key role in MMN generation [85]. The leading candidate mechanism studied in animal models is a phenomenon called stimulus-specific adaptation, in which neurons show sensitivity to the probability of sensory stimuli over many seconds, for example in oddball sequences [86, 87]. Stimulus-specific adaptation is more pronounced in cortical than subcortical auditory areas [88], and at least in the auditory cortex, appears to be driven not only by sensory adaptation to frequent stimuli but also by heightened sensitivity to infrequent stimuli (true deviance detection) [89]. However, most studies of stimulus-specific adaptation in auditory cortical neurons have not found the combination of long latency, deviance detection, and NMDA dependence that is characteristic of the human MMN [90, 91]. Stimulus-specific adaptation may therefore be an early auditory precursor rather than a direct neural correlate of the MMN.

Reduced MMN amplitude might also be a marker of genetic risk for psychosis. MMN amplitude is a heritable trait, with heritability estimates ranging from 58% to 68% [42, 92]. Two meta-analyses both reported suggestive evidence of a decrease in MMN amplitude among the unaffected first-degree relatives of patients with schizophrenia (Hedges’ *g* = −0.26 [71] and −0.21 [93]). These were trends rather than clearly significant differences probably due to the limited number of studies available for inclusion. A recent transcriptome-wide association study from our group found that MMN is associated with the expression of two genes (FAM89A and ENGASE) in the cortex, and genes associated with MMN were overexpressed in the frontal cortex during prenatal development but under-expressed in adulthood [94]. This study also compared the genetic overlap between schizophrenia and the MMN as well as two other biomarkers (verbal memory and ventricular volume), and concluded that the MMN was a superior candidate endophenotype due to its greater genetic overlap with the disease [94].

The MMN is a promising marker for early detection of individuals at clinical high risk for psychosis. A significant reduction of MMN amplitude was reported amongst high-risk individuals (Hedges’ *g* = −0.40) in the meta-analysis by Erickson et al. [71]. Furthermore, Bodatsch et al. conducted a systematic review and meta-analysis that compared individuals at clinical high risk who developed psychosis and those who did not [19]. They found that the duration MMN amplitude had the largest pooled effect size (Hedges’ *g* = −0.71) among all investigated EEG markers (including duration/frequency MMN, N100 difference/ratio, and P50 difference/ratio), although duration MMN had the most studies included (*n* = 5) thus with the greatest statistical power [19].

In conclusion, research on the MMN has been fruitful and it is a well-established biomarker for psychosis. A promising area for MMN research focuses on its neurobiology, especially regarding the role of NMDA receptors in MMN generation, and its underlying neural circuits in the context of predictive coding. The MMN holds potential for clinical applicability towards detecting those individuals at clinical high risk most likely to develop psychosis, who would benefit from prompt access to treatments. There has been less research on the MMN among unaffected relatives with mixed results, and more research is needed to clarify its genetic basis.

## The P300 waveform

The P300 is a positive voltage ERP, typically elicited by the auditory oddball paradigm [22, 95]. The P300 peak occurs ~300 ms following the presentation of a deviant (or target) stimulus which is randomly embedded in a series of frequent standard stimuli [20, 96, 97]. The P300 wave consists of two main sub-components occurring some 50 ms apart. The P3a is elicited by target stimuli that require no response from the participant and reflects attentional orienting and stimulus salience processing [20, 96, 97]. The following P3b is elicited by target stimuli requiring a response from the participant upon stimulus detection (for instance counting or pressing a button), and reflects working memory operations related to contextual updating [20, 96, 97].

Reduced P300 amplitude and prolonged P300 latency have consistently been found in patients with psychosis compared to healthy controls [22, 97–99]. A meta-analysis by our team comparing P300 between 1443 patients with schizophrenia and 1251 healthy controls reported a Cohen’s *d* of −0.85 for P300 amplitude and a Cohen’s *d* of 0.57 for P300 latency [22]. A more recent meta-analysis focusing on first-episode schizophrenia reported similar effect sizes (Cohen’s *d* = −0.83 for P300 amplitude and Cohen’s *d* = 0.48 for P300 latency) [99]. A meta-analysis of 30 studies with 1331 bipolar disorder patients (60% of whom had psychotic symptoms) and 1818 healthy controls reported that bipolar patients also exhibited reduced amplitude, and prolonged latency compared to the healthy subjects [97]. When examining only bipolar disorder with psychotic symptoms versus healthy controls, they found medium effect sizes with reduced amplitude (Hedges’ *g* = −0.58) and prolonged latencies (Hedges’ *g* = 0.52) [97].

The “context updating theory” postulates that the P300 amplitude reflects an attention-driven working memory comparison between new and repeated stimuli [20, 100]. Therefore, the more distinct the target is from the rest of the stimuli, the larger the P300 amplitude response will be. The P300 latency has been less precisely characterised but is thought to index processing speed, demonstrating how rapidly the individual responds to the stimulus, thus a neural correlate of reaction time [96]. Surprisingly, not many studies have reported the neuropsychological correlates of P300 in psychosis, except for some that found its association with memory performance [101, 102]. For clinical correlates, some studies reported negative correlations between P300 amplitude and symptom severity, especially for negative symptoms in psychosis [103, 104].

The P3a and P3b amplitudes show different topographic distributions, with P3a being prominent in the frontal lobes and P3b on the temporoparietal lobes [96, 97]. Given their different cortical sources, the P3a is hypothesised to be related to dopaminergic activity, while the P3b is related to norepinephrine activity [96, 100]. Indeed, some early pharmacological studies have found an effect of dopaminergic medications on the P300 (P3a) in humans [105, 106], although a later study using single-photon emission computerised tomography (SPECT) did not find any associations between dopamine transporter availability and P300 [107]. Besides, pharmacological studies using animal models also suggested an association between the locus coeruleus-norepinephrine system and the P300 (P3b) [108]. This is supported by a recent pilot study, which found that the P3b was influenced by transcutaneous vagus nerve stimulation (tVNS), a brain stimulation technique that may increase norepinephrine levels [109].

The P300 is one of the most well-established endophenotypes for psychosis, as similar deficits have been reported in the unaffected relatives of patients [93, 110, 111]. Heritability estimates are 68–80% for P300 amplitude and 21–56% for P300 latency [92]. When comparing unaffected relatives to controls, the latest meta-analyses reported a medium effect size (Hedges’ *g*) of −0.52 for P300 amplitude and 0.44 for P300 latency [93]. Malone et al. conducted a GWAS on P300 amplitude with 4211 participants, but found no hits that reached genome-wide significance, likely due to the limited sample size in a GWAS context [112]. Interestingly, their genome-wide analysis of all autosome genes identified an association with MYEF2 (myelin expression factor 2) [112], a promising finding awaiting replication.

Several studies described P300 deficits amongst individuals at clinical high risk compared to controls [95, 113–115]. A systematic review by Lepock et al. concluded that reduced P300 amplitude is a reliable finding in clinical high-risk individuals and thus has potential as a tool to identify people at risk [20]. However, fewer studies have compared the P300 between converters and non-converters within clinical high-risk individuals and the results were inconclusive [19, 70, 95]. In particular, a recent study by Tang et al. found that those people at clinical high risk who went on to develop psychosis had significantly lower P300 amplitude compared to healthy controls, and that high-risk cases who remitted had larger P300 amplitude compared to those remaining symptomatic or those who developed psychosis [95].

In summary, ample literature demonstrates that P300 deficits are evident not only in patients with psychosis, but also in their unaffected relatives and individuals at clinical high risk, thus, rendering P300 a correlate of genetic and clinical vulnerability to psychosis. However, despite the extensive literature on the P300 in psychosis, there has been limited research on its neuropsychological correlates and underlying neurobiology. Future pharmacological experiments across species, as well as studies employing other imaging methods and genomics, should help to address these gaps. The P300 could also have value for screening individuals at high risk of developing psychosis, although more data from longitudinal studies are needed to establish its potential for clinical applications.

## Other neurophysiological markers for psychosis

### N100 in corollary discharge

The N100 has also been used to measure deficits in corollary discharge in psychosis using the talk-listen paradigm. The corollary discharge theory contends that when individuals initiate an action, they also generate an ‘efferent copy’ of the same action in the brain [116]. The brain compares the actual and expected outcomes based on the efferent copy and suppresses the following response if the two outcomes are identical, which is referred to as ‘corollary discharge’ [116]. Patients with psychosis are hypothesised to have deficits in corollary discharge, and thus fail to suppress self-generated sensations. In the talk-listen paradigm, participants’ N100 responses are compared during listening to external stimuli and during their own speech. Several studies have found that patients with psychosis did not suppress N100 responses during their own speech to the same extent as controls [116–118]. As a theory-based approach, the N100 in the context of corollary discharge could illuminate the neurobiology of psychosis symptoms especially auditory hallucinations.

### N400

The N400 is a negative voltage ERP elicited by stimuli that violate the semantic context, thus exploring language as a key skill frequently affected in psychosis. It is often measured using picture–word matching tasks [119]. Words that do not match preceding pictures trigger a larger N400 wave than the congruent word–picture pairs [119]. Previous studies have consistently found that patients with psychosis showed reduced N400 amplitude to semantically incongruent words compared to controls, as well as smaller priming effects (difference between N400 amplitude to congruent and incongruent words) [119, 120]. Similar deficits in N400 were also found in individuals at clinical high risk [121], but not in unaffected relatives of patients [122, 123], indicating that N400 might be a state marker for psychosis instead of an endophenotype of genetic predisposition.

### Neural oscillations

In addition to ERPs, neural oscillations are also attractive biomarkers for psychosis due to their mapping into underlying local neural circuits and great transferability across species [124]. Deficits in gamma oscillations, measured by both evoked-activity paradigms and the auditory steady-state response paradigm, remain one of the most researched and well-replicated impairments in psychosis [125]. Abnormal gamma oscillations in psychosis are thought to be associated with the dysfunction of parvalbumin interneurons [126], which underlie gamma abnormalities in rodent models of NMDA receptor dysfunction [127]. By contrast, there has been conflicting evidence for abnormal neural oscillations in psychosis measured during resting state [125], and less research is available on lower-frequency oscillations. Future research should use animal models to elucidate the nature of such changes and help to identify new treatment targets.

### Pre-pulse inhibition

Electromyography (EMG) has been used extensively to measure pre-pulse inhibition (PPI) in psychosis research. PPI is a marker of sensorimotor gating, referring to the attenuated amplitude of the startle response when a weak pre-stimulus is administered prior to a loud startling stimulus [128, 129]. Converging evidence in the literature shows that patients with psychosis exhibit impaired PPI compared to controls. A meta-analysis of 67 studies with a total of 4290 people with schizophrenia and 3685 healthy controls reported a lower level of PPI in the patient group with medium effect sizes (Hedges’ *g*) ranging from −0.50 to −0.44, depending on the interstimulus interval [130]. Two recent meta-analyses did not find evidence for PPI deficits in first-degree relatives of patients with psychosis [128], but found that individuals at clinical high risk had reduced PPI with an effect size (Cohen’s *d*) of −0.62 [129]. PPI deficits in psychosis are thought to share similar mechanisms with P50 and N100 sensory gating and are considered biomarkers of sensory information overload.

## Discussion

The current review summarised the evidence for neurophysiological deficits in psychosis, as well as their potential research and clinical implications. There is strong evidence supporting deficits in P50 sensory gating, N100 amplitude (at long interstimulus intervals), MMN amplitude, and P300 amplitude/latency amongst patients with psychosis, rendering them neurophysiological biomarkers for the disease. Some of the neurophysiological markers may indicate the genetic or clinical risk of psychosis: although only deficits in P300 amplitude and latency have been established in both unaffected relatives and individuals at clinical high risk, there is also evidence supporting P50 gating impairment in unaffected relatives and MMN impairment in clinical high-risk individuals.

As EEG is a direct in vivo measure of neuronal electrical activity related to information processing, neurophysiological markers for psychosis are key to elucidate disease mechanisms. MMN and P300 are both related to the detection of deviant auditory stimuli. MMN explores the early pre-conscious stages of perception in the auditory cortex, while P300 captures higher-order cognitive processing including memory and attention. Although N100, MMN, and P300 can all be reliably measured by the auditory oddball paradigm, Rissling and Light proposed that they may represent three different underlying processes: sensory registration, automatic change detection, and attentional orientation/allocation [131]. Another group of neurophysiological markers, including P50 sensory gating, N100 sensory gating, and PPI, arise from other perceptual processes such as sensory adaptation and sensorimotor inhibition [132]. However, there has only been limited research investigating their clinical and neuropsychological correlates, and more work is needed to clarify the distinct and overlapping mechanisms between those biomarkers. Besides, while many hypotheses have been proposed, we need more compelling evidence to understand their neurobiology. Since many neurophysiological markers are comparable across species, translational and pharmacological studies using animal models are particularly useful to identify the underlying neural circuits and neurotransmitters. Other imaging methods, such as magnetoencephalography (MEG), SPECT, functional/structural magnetic resonance imaging (MRI), and non-invasive neurostimulation, should also be employed in conjunction with EEG to elucidate disease mechanisms in psychosis.

**Fig. 1:**
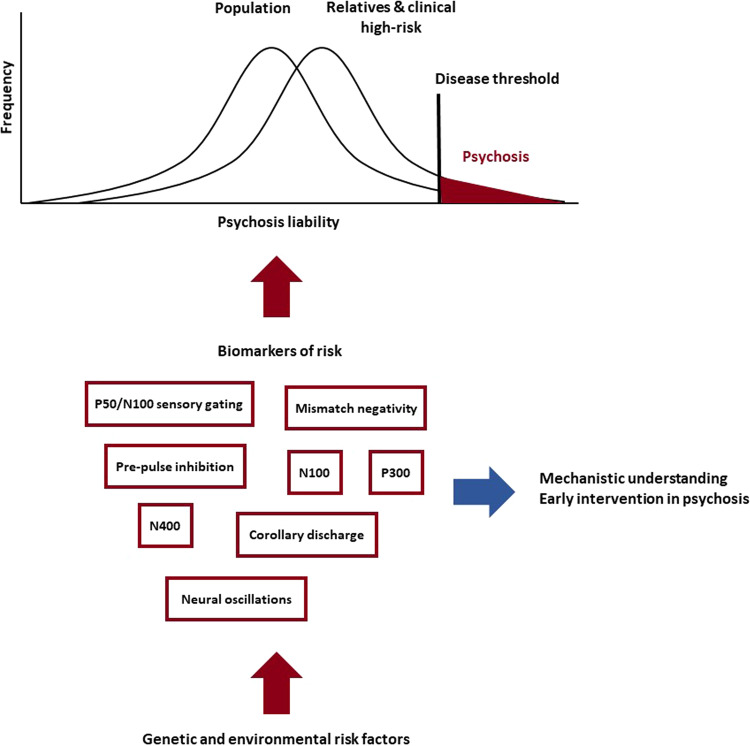
The utility of neurophysiological markers for psychosis in the multifactorial threshold model [133, 134]. Neurophysiological deficits found amongst unaffected relatives can serve as biomarkers of genetic risk to identify relevant biological mechanisms, while neurophysiological tests in high-risk individuals have potential to aid clinical practice in early detection and treatments for psychosis.

Figure 1 illustrates the utility of neurophysiological markers for psychosis in the multifactorial threshold model [133, 134]. In addition to deficits observed in patients, neurophysiological markers of genetic and clinical risk of psychosis can also improve our mechanistic understanding and potentially aid clinical practice. Genetic advances offer unprecedented insights into the aetiology of psychosis, yet imaging tools such as neurophysiological biomarkers of genetic risk (endophenotypes) are also key to elucidate disease mechanisms. As reviewed above, the classical method for testing the appropriateness of a potential endophenotype is through family studies that involve the unaffected relatives of people with psychosis [18], which are still needed for some markers, such as the N100. Another novel approach involves using polygenic risk scores to investigate if a neurophysiological marker is associated with common genetic variants increasing psychosis risk [135]. Once a biomarker has been established as an endophenotype, traditional methods such as candidate-gene and linkage analysis [43–45], as well as novel and hypothesis-free methods such as genome-wide or transcriptome-wide association studies [94, 112] and sequencing technologies can help identify its relevant genetic variants and biological pathways, which may serve as potential treatment targets. On the other hand, biomarkers of the clinical high-risk state could facilitate the early detection of those individuals, and thus accelerate access to timely treatments. While deficits in some markers have been well established in clinical high-risk populations (e.g. MMN amplitude, P300 amplitude/latency), others still await further research (e.g. P50 sensory gating, N100 amplitude). Previous research also suggests that about one in three individuals at clinical high risk will develop psychosis, while others remain at clinical high risk or even remit [136]. Therefore, identifying simple biological markers and tests that can help clinicians identify those people most likely to transition to psychosis before severe symptoms emerge is crucial. The neurophysiology literature in this field is promising but currently scarce.

It is worth noting potential confounders, particularly antipsychotic medications, in case-control studies. The vast majority of neurophysiological studies involved participants taking antipsychotics. Although medication-free patients with first-episode psychosis exhibit similar neurophysiological deficits to chronic patients, such deficits are usually reported to be milder [71, 93]. Indeed, a systematic review and meta-analysis by Jackson and Seneviratne found that some antipsychotics, in particular clozapine, were associated with EEG slowing and epileptiform discharges (odds ratios = 16.9 and 6.2, respectively) [137]. Surprisingly, only a few studies have investigated the effect of antipsychotics on ERPs, mostly on small samples [138–144]. This highlights the importance of research in medication-free participants, as well as studies monitoring patients’ EEG or ERP changes before and after taking medication. Furthermore, there is substantial methodological heterogeneity in neurophysiological research in psychosis. The same EEG or ERP marker could be elicited using diverse paradigms and analysed by different methods. Methodological heterogeneity may also explain why there have been fewer systematic reviews or meta-analyses on certain markers, such as N100 and neural oscillations. Such evidence in previous literature still needs to be summarised systematically, and standardised paradigms and analysis pipelines would benefit future studies.

To conclude, EEG and ERPs are promising due to their ubiquity, temporal resolution and capacity for cross-species insights into neurobiology. Although there is evidence for deficits in P50 sensory gating, N100 amplitude, MMN amplitude, and P300 amplitude/latency in patients with psychosis, we need more studies to validate their role as markers for psychosis risk amongst unaffected relatives and individuals at clinical high risk. The combined use of neurophysiological markers and genetics holds great potential to illuminate the aetiology of psychosis and facilitate the development of new drugs, while research in high-risk individuals could allow more rapid access to psychological and medical treatments for people with psychosis.
